# Supplementary material for: Genome-Wide Identification, Characterization, and Expression Profiling of AP2/ERF Superfamily Genes under Different Development and Abiotic Stress Conditions in Pecan (Carya illinoinensis)
Source: Int J Mol Sci. 2022 Mar 8;23(6):2920. doi: 10.3390/ijms23062920 (PMC8950532; doi:10.3390/ijms23062920)
Supplement: Supplementary file 1 [file ijms-23-02920-s001.zip › Figures S1 and S2.pdf]

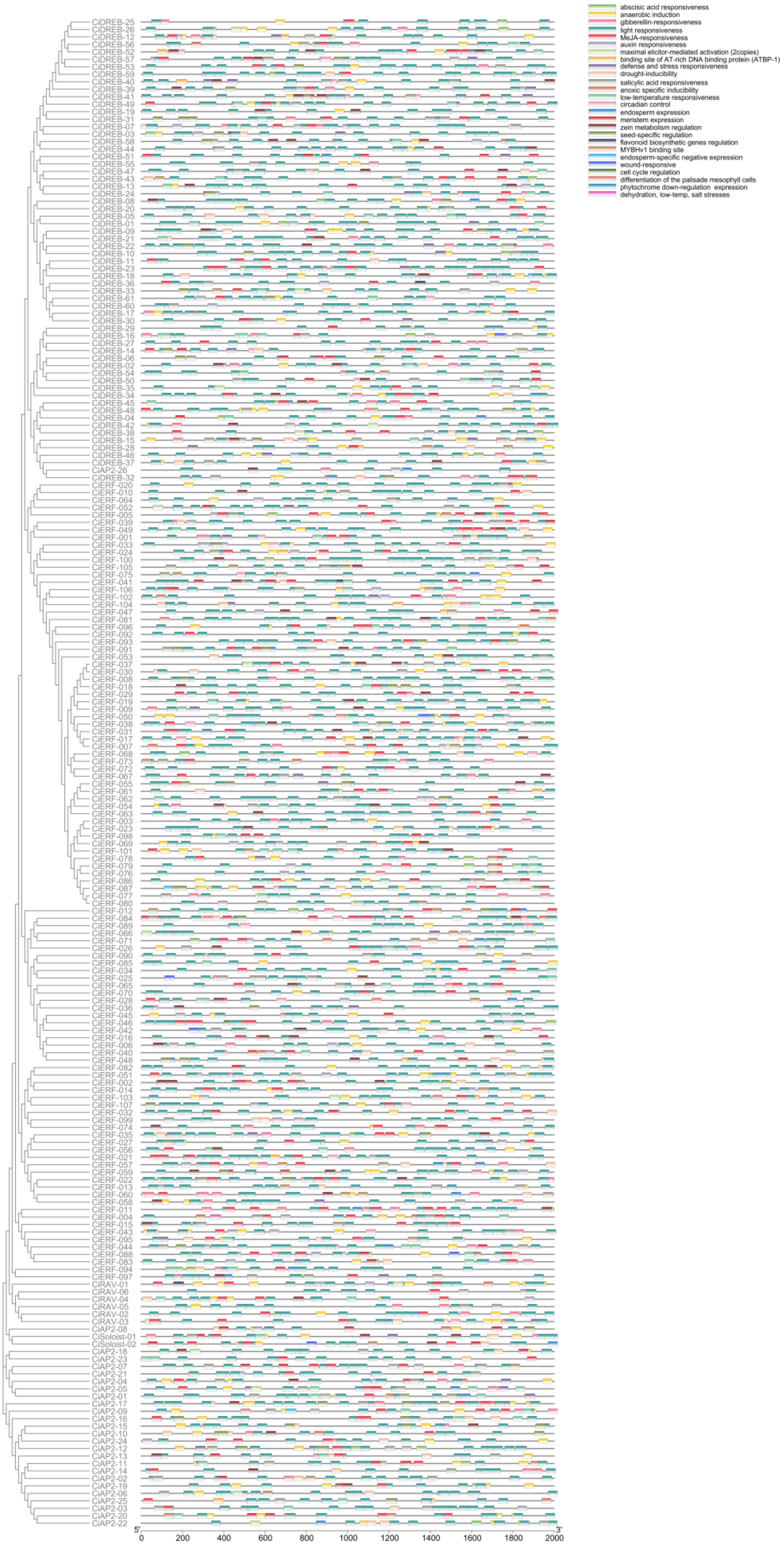

abscisic acid responsiveness
anaerobic induction
gibberellin-responsiveness
light responsiveness
MeJA-responsiveness
auxin responsiveness
maximal elicitor-mediated activation (2copies)
binding site of AT-rich DNA binding protein (ATBP-1)
defense and stress responsiveness
drought-inducibility
salicylic acid responsiveness
anoxic specific inducibility
low-temperature responsiveness
circadian control
endosperm expression
meristem expression
zein metabolism regulation
seed-specific regulation
flavonoid biosynthetic genes regulation
MYBHv1 binding site
endosperm-specific negative expression
wound-responsive
cell cycle regulation
differentiation of the palisade mesophyll cells
phytochrome down-regulation expression
dehydration, low-temp, salt stresses
CiDREB-25
CiDREB-26
CiDREB-12
CiDREB-56
CiDREB-52
CiDREB-57
CiDREB-53
CiDREB-59
CiDREB-40
CiDREB-39
CiDREB-41
CiDREB-49
CiDREB-19
CiDREB-31
CiDREB-07
CiDREB-03
CiDREB-58
CiDREB-44
CiDREB-51
CiDREB-55
CiDREB-47
CiDREB-43
CiDREB-13
CiDREB-24
CiDREB-08
CiDREB-20
CiDREB-05
CiDREB-01
CiDREB-09
CiDREB-21
CiDREB-22
CiDREB-10
CiDREB-11
CiDREB-23
CiDREB-18
CiDREB-36
CiDREB-33
CiDREB-61
CiDREB-60
CiDREB-17
CiDREB-30
CiDREB-29
CiDREB-16
CiDREB-27
CiDREB-14
CiDREB-06
CiDREB-02
CiDREB-54
CiDREB-50
CiDREB-35
CiDREB-34
CiDREB-45
CiDREB-48
CiDREB-04
CiDREB-42
CiDREB-38
CiDREB-15
CiDREB-28
CiDREB-46
CiDREB-37
CiAP2-26
CiDREB-32
CiERF-020
CiERF-010
CiERF-064
CiERF-052
CiERF-005
CiERF-039
CiERF-049
CiERF-001
CiERF-033
CiERF-024
CiERF-100
CiERF-105
CiERF-075
CiERF-041
CiERF-106
CiERF-102
CiERF-104
CiERF-047
CiERF-081
CiERF-096
CiERF-092
CiERF-093
CiERF-091
CiERF-053
CiERF-037
CiERF-030
CiERF-008
CiERF-018
CiERF-029
CiERF-019
CiERF-009
CiERF-050
CiERF-038
CiERF-031
CiERF-017
CiERF-007
CiERF-068
CiERF-073
CiERF-072
CiERF-067
CiERF-055
CiERF-061
CiERF-062
CiERF-054
CiERF-063
CiERF-003
CiERF-023
CiERF-098
CiERF-069
CiERF-101
CiERF-078
CiERF-079
CiERF-076
CiERF-086
CiERF-087
CiERF-077
CiERF-080
CiERF-012
CiERF-084
CiERF-089
CiERF-066
CiERF-071
CiERF-026
CiERF-090
CiERF-085
CiERF-034
CiERF-025
CiERF-065
CiERF-070
CiERF-028
CiERF-036
CiERF-045
CiERF-046
CiERF-042
CiERF-016
CiERF-006
CiERF-040
CiERF-048
CiERF-082
CiERF-051
CiERF-002
CiERF-014
CiERF-103
CiERF-107
CiERF-032
CiERF-099
CiERF-074
CiERF-035
CiERF-027
CiERF-056
CiERF-021
CiERF-057
CiERF-059
CiERF-022
CiERF-013
CiERF-060
CiERF-058
CiERF-011
CiERF-004
CiERF-015
CiERF-043
CiERF-095
CiERF-044
CiERF-088
CiERF-083
CiERF-094
CiERF-097
CiRAV-01
CiRAV-06
CiRAV-04
CiRAV-05
CiRAV-02
CiRAV-03
CiAP2-08
CiSoloist-01
CiSoloist-02
CiAP2-18
CiAP2-23
CiAP2-07
CiAP2-21
CiAP2-04
CiAP2-05
CiAP2-01
CiAP2-17
CiAP2-09
CiAP2-16
CiAP2-15
CiAP2-10
CiAP2-24
CiAP2-12
CiAP2-13
CiAP2-11
CiAP2-14
CiAP2-02
CiAP2-19
CiAP2-06
CiAP2-25
CiAP2-03
CiAP2-20
CiAP2-22
5'
3'
0
200
400
600
800
1000
1200
1400
1600
1800
2000

Figure S1. Schematic representations of cis-regulatory elements present in *AP2/ERF*s as investigated via the PlantCARE tool.

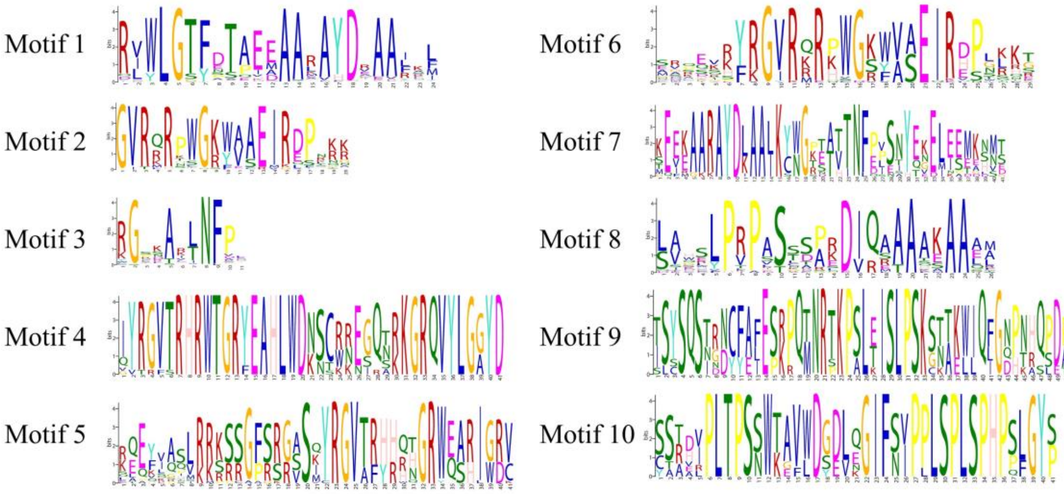

Figure S2. Detailed information of motifs
